# Immune-Related Molecules *CD3G* and *FERMT3*: Novel Biomarkers Associated with Sepsis

**DOI:** 10.3390/ijms25020749

**Published:** 2024-01-06

**Authors:** Nanxi Li, Peng Ren, Jingya Wang, Xiaohui Zhu, Xuan Qiao, Zhirui Zeng, Tong Ye, Shanshan Wang, Zhiyun Meng, Hui Gan, Shuchen Liu, Yunbo Sun, Xiaoxia Zhu, Guifang Dou, Ruolan Gu

**Affiliations:** 1 Department of Pharmaceutical Sciences, Beijing Institute of Radiation Medicine, Beijing 100850, China; 2 Beijing Institute of Basic Medical Sciences, Beijing 100850, China; 3 Guizhou Provincial Key Laboratory of Pathogenesis & Drug Research on Common Chronic Diseases, Department of Physiology, School of Basic Medical Sciences, Guizhou Medical University, Guiyang 550000, China

**Keywords:** sepsis, hub gene, biomarkers, CD3G, FERMT3, WGCNA

## Abstract

Sepsis ranks among the most common health problems worldwide, characterized by organ dysfunction resulting from infection. Excessive inflammatory responses, cytokine storms, and immune-induced microthrombosis are pivotal factors influencing the progression of sepsis. Our objective was to identify novel immune-related hub genes for sepsis through bioinformatic analysis, subsequently validating their specificity and potential as diagnostic and prognostic biomarkers in an animal experiment involving a sepsis mice model. Gene expression profiles of healthy controls and patients with sepsis were obtained from the Gene Expression Omnibus (GEO) and analysis of differentially expressed genes (DEGs) was conducted. Subsequently, weighted gene co-expression network analysis (WGCNA) was used to analyze genes within crucial modules. The functional annotated DEGs which related to the immune signal pathways were used for constructing protein–protein interaction (PPI) analysis. Following this, two hub genes, *FERMT3* and *CD3G*, were identified through correlation analyses associated with sequential organ failure assessment (SOFA) scores. These two hub genes were associated with cell adhesion, migration, thrombosis, and T-cell activation. Furthermore, immune infiltration analysis was conducted to investigate the inflammation microenvironment influenced by the hub genes. The efficacy and specificity of the two hub genes were validated through a mice sepsis model study. Concurrently, we observed a significant negative correlation between the expression of CD3G and IL-1β and GRO/KC. These findings suggest that these two genes probably play important roles in the pathogenesis and progression of sepsis, presenting the potential to serve as more stable biomarkers for sepsis diagnosis and prognosis, deserving further study.

## 1. Introduction

Sepsis is a life-threatening infection which can cause inflammation storm and disseminated intravascular coagulation, making it a leading cause of death among intensive care unit (ICU) patients [1,2]. Recent global estimates for sepsis incidence and mortality reported 19.4 million sepsis incident cases and 5.3 million sepsis-related deaths annually [3]. However, diagnosing and evaluating sepsis is challenging due to the highly variable and nonspecific nature of its signs and symptoms. For example, the overall sensitivity of the systemic inflammatory response syndrome (SIRS) criteria in detecting sepsis is only about 50% to 60% and that of the quick version of sequential organ failure assessment (qSOFA) is approximately 50% [4]. Current diagnosis of sepsis primarily relies on physiological and biochemical indexes and clinical manifestations, such as physical signs, symptoms of multiple organ failure, and inflammatory factor detection. However, it lacks the assessment of upstream immune system molecules to evaluate the immune system. Consequently, this deficiency renders the sequential organ failure assessment (SOFA) score assay relatively insufficient in terms of timeliness and specificity for assessing organ dysfunction and predicting ICU mortality, in the context of the Third International Consensus Definitions for Sepsis and Septic Shock (sepsis-3.0), announced at the 45th Critical Care Congress [2,5].

Currently, a variety of biomarkers are employed to aid in the clinical diagnosis of sepsis, including C-reactive protein, P-selection, vascular cell adhesion molecule-1, programmed death-1, interleukin 6, interleukin 10, etc. [6]. Immunocytes play key roles in combating pathogens via releasing inflammation cytokines. However, the consequence of an excessively activated immune system primarily manifests as disturbing the balance between pro-inflammation and anti-inflammation, thereby promoting the progression of sepsis [7,8]. Previous research showed that neutrophils and macrophages presented dysfunction during sepsis, characterized by extensive infiltration into the tissues and chemotaxis [9]. For instance, excessive neutrophil migration during the early stages of sepsis may lead to exaggerated inflammatory response with associated tissue damage and subsequent organ dysfunction [10]. The autopsies of sepsis patients also showed that immune cell death is one of the causes of the mortality [11], all of which indicates that these immune-related signatures may be of great importance in sepsis diagnosis.

The primary challenge associated with biomarkers for sepsis revolves around their insufficient specificity, attributable to the intricate pathogenesis and diverse clinical manifestations of sepsis. Currently, the majority of existing biomarkers of sepsis are downstream molecules in the signaling pathway, such as pro-inflammatory factors, cytokines, and chemokines. These molecules are susceptible to various interfering factors during their expression process, leading to compromised specificity and stability. Therefore, searching for upstream immune-related molecules is anticipated to enhance the specificity and stability of diagnostic and predictive biomarkers for sepsis. This approach not only holds the potential to advance early diagnosis and prediction but also to contribute to improve the implementation strategies for sepsis therapeutics [12]. Since it may provide earlier predictions of the precise progression of sepsis based on the upstream biomarker involved, clinicians can implement targeted adjustments to treatment plans in time.

Bioinformatic analysis is a valuable and effective approach for investigating novel biomarkers associated with various diseases as well as assessing the extent of immunocytes infiltration. Numerous studies have employed this method to explore potential hub genes, enabling the prediction of disease progression [13]. Additionally, bioinformatics can serve as a novel tool for identifying biomarkers of sepsis, and the upstream gene markers obtained through bioinformatics methods can serve as an important auxiliary tool in diagnosing and predicting the progression and prognosis of sepsis, as well as enabling timely adjustment of treatment strategies. Dai W. et al. demonstrated the involvement of the ferroptosis-related gene LPIN1 in inflammation and infection during sepsis. They utilized bioinformatic analysis to screen key genes, subsequently validating their findings in CLP rat models, as well as LPS-induced rat lung macrophage NR8383 cells and rat lung epithelial type II RLE-6TN cells [14]. In a study by Xu C, et al., the focus was on immune infiltration in sepsis, leading to the identification of CEBPB as a potential biomarker [15]. Meanwhile, Godini R. et al. uncovered the system-level responses of neutrophils and peripheral blood mononuclear cells to sepsis, identifying 11 potential genes for therapeutic and diagnostic purposes [16]. To accurately identify novel immune-related biomarkers for early diagnosis and prediction of sepsis, we employed a bioinformatics algorithm combined with the SOFA score. This approach allowed us to analyze gene expression changes between septic patients and healthy individuals, leading to the successful identification of two hub genes, *CD3G* and *FERMT3*. Notably, CD3G is associated with T-cell activation, while FERMT3 is linked to cell adhesion, migration, and thrombosis. Subsequently, we verified the expression changes of these two genes in sepsis mice and their correlation with inflammatory factor levels through animal experiments.

## 2. Results

### 2.1. Data Processing and Identification of Differentially Expressed Genes (DEGs)

**Figure 1:**
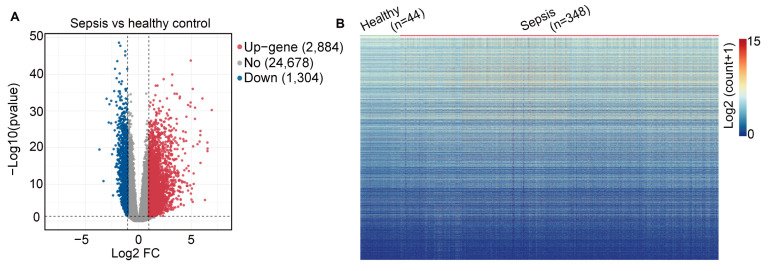
Identification of DEGs in sepsis. (**A**) Volcano plot showing differentially expressed genes between sepsis and healthy groups. (**B**) Heat map indicating DEGs between sepsis and healthy groups in peripheral blood.

The GSE185263 dataset was obtained from GEO database (https://www.ncbi.nlm.nih.gov/gds accessed on 10 February 2023), comprising 44 healthy samples and 348 sepsis samples. DEGs were identified using R software (version: 3.5.2), with the cutoff criteria set as |log2FC| ≥ 2 and an adjusted *p*-value < 0.05. The volcano plot illustrates the presence of 4188 genes with differential expression profiles, including 2884 up-regulated genes and 1304 down-regulated genes. Figure 1A,B displays a heat map indicating DEGs between the sepsis and healthy groups in peripheral blood. After excluding non-differentially expressed genes, the genuine DEGs were isolated and subsequently included in the weighted correlation network analysis (WGCNA). As shown in Figure S2, the heat map was generated using the top ten up-regulated and down-regulated genes to support our identification of DEGs.

### 2.2. Filtering the Gene Modules by Constructing WGCNA

**Figure 2:**
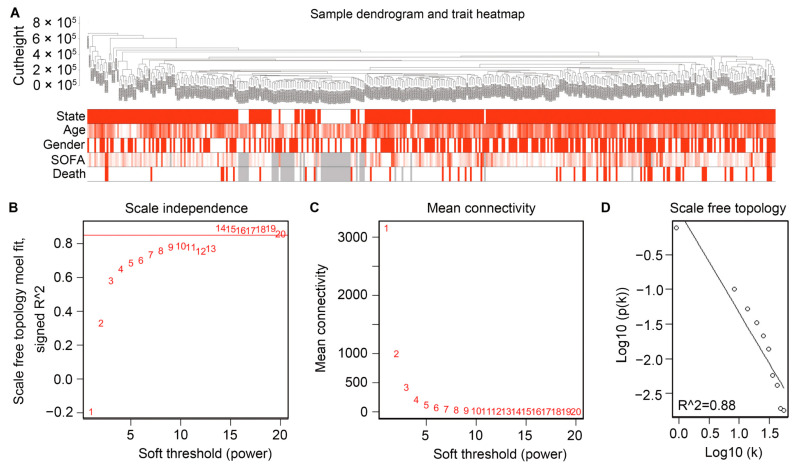
Construction of WGCNA and analysis of the network topology for various soft-threshold powers. (**A**) Sample dendrogram and trait heat map. In the state module, the red color represents sepsis patients, while the white color represents healthy controls; In the age module, the intensity of red color indicates the size of age; In the gender module, the red color represents males, while the white color represents females; In the SOFA module, the intensity of the red color indicates the level of organ dysfunction; In the death module, the red color represents the fatal outcome, while the white color represents survival. The missing data were labeled in gray. (**B**) Scale-free fit parameters of different soft-thresholding powers. (**C**) Average connectivity of different soft-thresholding powers. (**D**) Scale-free topology.

The gene expression data from peripheral blood specimens of patients who suffered from sepsis (*n* = 348) and healthy individuals (*n* = 44), along with clinical phenotype data, were employed for WGCNA. A hierarchical clustering tree was constructed through dynamic hybrid cutting. Each leaf on the tree represents the gene expression signature of a single patient, and genes with similar expression levels are adjacent, forming a branch of the tree, referred to as a gene module. The underlying topology of biological networks is approximately “scale-free” [17]. When the β value was set to 14 as the appropriate soft-threshold power, the topological overlap matrix met the standards for scale-free topology. The scale independence was >0.80, accompanied by mean connectivity verging on 0 (Figure 2B–D) [18]. Thus, the network constructed with β = 14 adheres to the power-law distribution and a better approximation of the real biological network. Ultimately, 15 modules were generated, and no outliers were identified through the sample dendrogram. The height of all samples was <8 × 10^5^ (Figure 2A).

**Figure 3:**
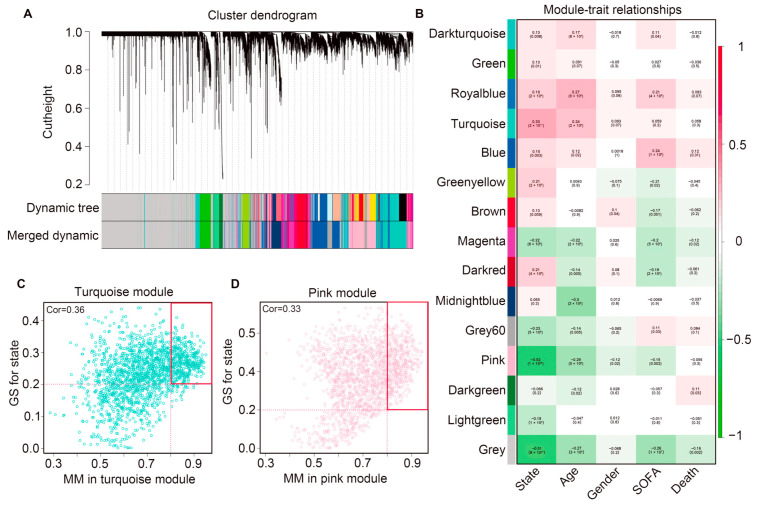
Conducted weighted gene co-expression network analysis. (**A**) Modules were identified in the resulting dendrogram using the dynamic tree. (**B**) The significant modules associated with the clinical traits. A total of 15 modules were identified. Modules were distinguished from each other by assigning different colors. Each cell in the heat map contains the corresponding correlation score and *p*-value between gene modules and clinical traits. Red indicates positive correlation and green indicates negative correlation. (**C**,**D**) Relationship between GS and MM in the significant modules. Both two red boxes represent module core genes which collected based on the screening criteria (MM > 0.8 and GS > 0.2) from the turquoise and pink models. A total of 845 module core genes were selected.

Gene dendrograms were prepared, and genes were clustered into modules according to the interconnections between gene pairs. Modules in this context refer to clusters of densely interconnected genes [19]. Such modules often exhibit a pronounced enrichment of functional classes [20]. There were 15 gene co-expression modules (dark turquoise, green, royal blue, turquoise, blue, green-yellow, brown, magenta, dark red, midnight blue, grey60, pink, dark green, light green, gray) identified. Genes lacking co-expression relationships were selectively clustered in the gray module (Figure 3A,B). Moreover, we specifically determined the turquoise and pink gene modules for their correlation with clinical characteristics in sepsis samples, aiming to establish a close association between these modules and patients with sepsis. The turquoise module showed a positive correlation with the state of samples (R = 0.33, *p* = 2 × 10^−11^) while the pink module exhibited a negative correlation with the state of samples (R = −0.52, *p* = 1 × 10^−28^).

To select genes that were highly consistent with clinical state within the selected two modules, we calculated the correlations between gene significance (GS) and module membership (MM). In particular, a higher GS value indicated greater clinical significance of the genes. The MM of the turquoise module exhibited a correlation with the GS for clinical state (correlation = 0.36, *p* < 0.05) and similarly, the MM of the pink module showed a correlation with the GS for clinical state (correlation = 0.33, *p* < 0.05) (Figure 3C,D).

### 2.3. Functional and Pathway Enrichment Analysis for Hub Genes

**Figure 4:**
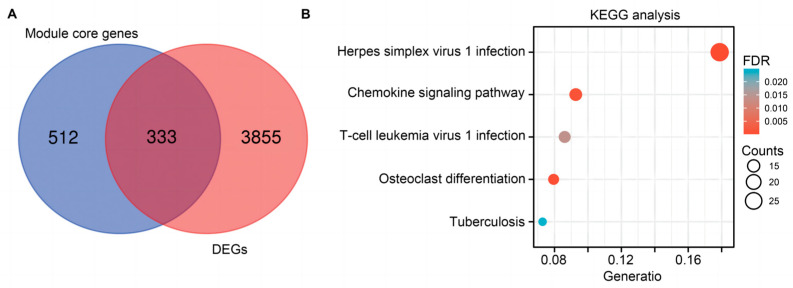
Functional enrichment analysis of DEGs. (**A**) Venn diagram of module core genes and DEGs by intersection analysis. (**B**) The bubble plot of KEGG pathway analysis results. Enriched terms in KEGG pathway analysis *p* < 0.05. The count value presents the number of genes in the KEGG pathway from overlap genes.

The Venn diagram showed that 333 genes were identified through interaction analysis among 3855 DEGs and 845 module core genes, collected based on the screening criteria (MM > 0.8 and GS > 0.2) from the turquoise and pink models. Subsequently, Kyoto Encyclopedia of Genes and Genomes (KEGG) pathway analysis was employed to functionally annotate these genes. The results indicated that the core genes may play a pivotal role in immune-related pathways and immune cell infiltration during sepsis. Noteworthy pathways included herpes simplex virus 1 infection, chemokine signaling pathway, T-cell leukemia virus 1 infection, and osteoclast differentiation (Figure 4A,B).

### 2.4. PPI Network Construction

**Figure 5:**
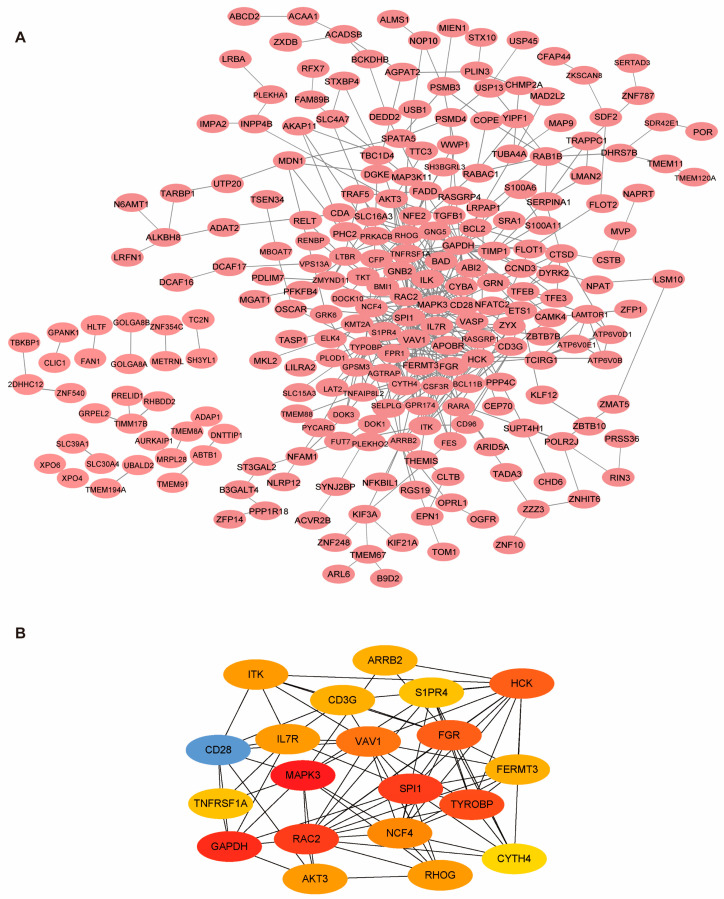
Hub gene selection by PPI analysis. (**A**) PPI networks were constructed using the STRING tool at a median confidence interval of 0.400, while edges indicate the correlation between core genes. (**B**) Cytoscape was used to analyze the PPI network. Core genes were selected based on the connection degree within the PPI subnetwork.

The PPI network, featuring a confidence score exceeding 0.4 for the 333 core genes, was visualized using Cytoscape software (version: 3.7.2) (Figure 5A). Analyzing the connection degree scores within this PPI network, key genes with degree of connection degree (top 10) were identified and designated as hub genes, including IL2 inducible T cell kinase (*ITK*), arrestin beta 2 (*ARRB2*), *HCK* proto-oncogene, Src family tyrosine kinase (*HCK*), *CD3* gamma subunit of T-cell receptor complex (*CD3G*), sphingosine-1-phosphate receptor 4 (*S1PR4*), *CD28* molecule (*CD28*), interleukin 7 receptor (*IL7R*), vav guanine nucleotide exchange factor 1 (*VAV1*), *FGR* proto-oncogene, Src family tyrosine kinase (*FGR*), FERM domain containing kindlin 3 (*FERMT3*), TNF receptor superfamily member 1A (*TNFRSF1A*), mitogen-activated protein kinase 3 (*MAPK3*), Spi-1 proto-oncogene (*SPI1*), transmembrane immune signaling adaptor (*TYROBP*), cytohesin 4 (*CYTH4*), ras homolog family member G (*RHOG*), neutrophil cytosolic factor 4 (*NCF4*), Rac family small GTPase 2 (*RAC2*), AKT serine/threonine kinase 3 (*AKT3*), glyceraldehyde-3-phosphate dehydrogenase (*GAPDH*) (Figure 5B). These genes highly correlate with the immune system and accord with the results from KEGG analysis.

### 2.5. Gene Set Enrichment Analysis (GSEA) Analysis of Two Hub Genes Revealed Their Involvement of the Immune System

**Figure 6:**
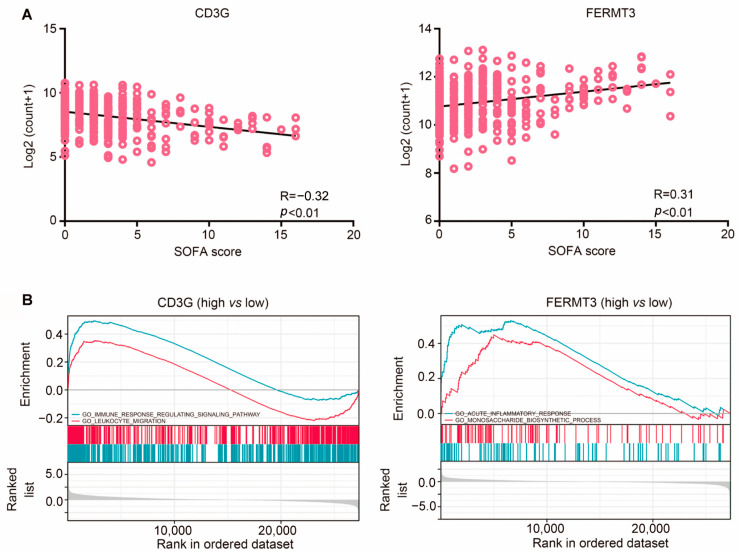
Correlation between hub genes and SOFA score and functional analyses of the key genes by GSEA and GO. (**A**) *CD3G* and *FERMT3* were selected out from 20 core genes based on the SOFA score. (**B**) GO functional enrichment and GSEA analysis, including *CD3G* and *FERMT3*, revealed the underlying immune related functions of up- and down-regulated hub genes. (False discovery rate (FDR) < 0.05 was present if a set was significantly enriched).

Two hub genes were identified by utilizing correlation analysis between SOFA score and gene expression levels. The analysis revealed a negative correlation between the expression of *CD3G* and the SOFA score, while *FERMT3* exhibited a positive correlation with the SOFA score according to Pearson analysis. Subsequently, gene ontology (GO) enrichment analysis highlighted that the functions of CD3G were predominantly associated with leukocyte migration, whereas FERMT3 was concentrated in acute inflammatory response and monosaccharide biosynthetic processes. GSEA was performed to predict the regulated mechanism and biological roles of *CD3G* and *FERMT3* based on their high and low expression levels of these two hub genes. This method functions by focusing on gene sets which are the groups of genes that share common biological function and regulation [21]. The results indicated that both *CD3G* and *FERMT3* play crucial roles in the immune system during sepsis (Figure 6A,B).

### 2.6. Two Hub Genes Involved in Immune Cell Infiltration

**Figure 7:**
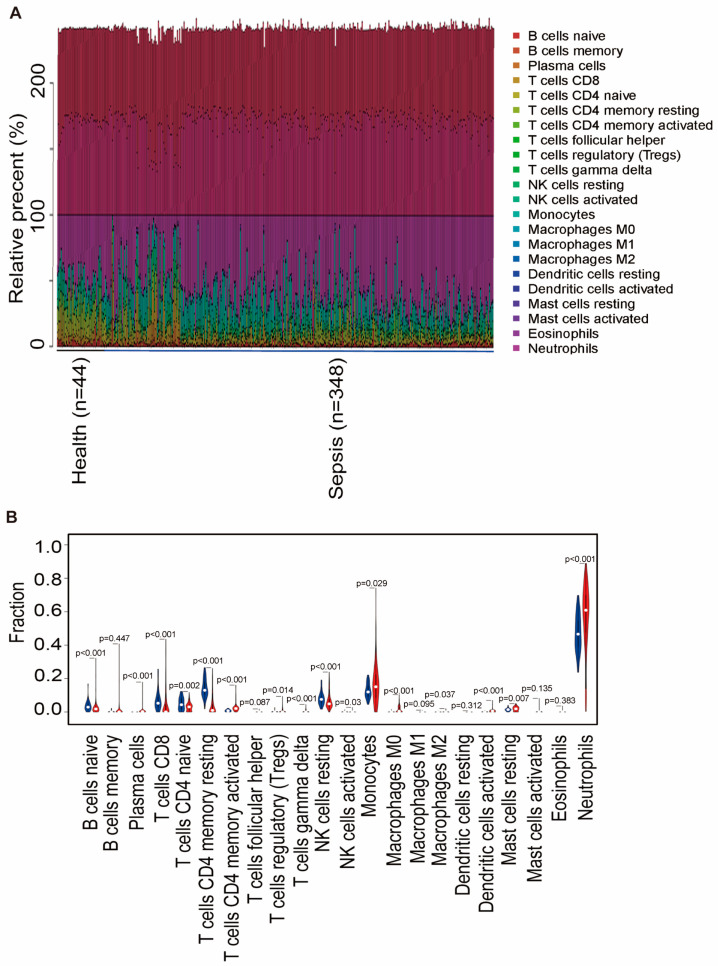
Landscape of immune cell infiltration in healthy and sepsis groups. (**A**) Relative percentage of 22 subpopulations of immune cells in the samples. (**B**) Difference in immune infiltration between the healthy and sepsis groups. (Healthy group marked in blue; sepsis group marked in red). *p*-values < 0.05 were considered statistically significant.

The differences in immune infiltration between the sepsis and control tissue samples were examined across 22 subpopulations of immune cells using the Cibersort method (Figure 7A). The violin plot of the differences in immune cell infiltration revealed heightened levels of activated CD4 memory T cells, monocytes, macrophages, activated dendritic cells, resting mast cells, and neutrophils compared with those observed in the healthy control samples (Figure 7B). These findings strongly suggest that the inflammatory microenvironment plays a pivotal role in reshaping the proportion and distribution of immune cells in patients with sepsis.

**Figure 8:**
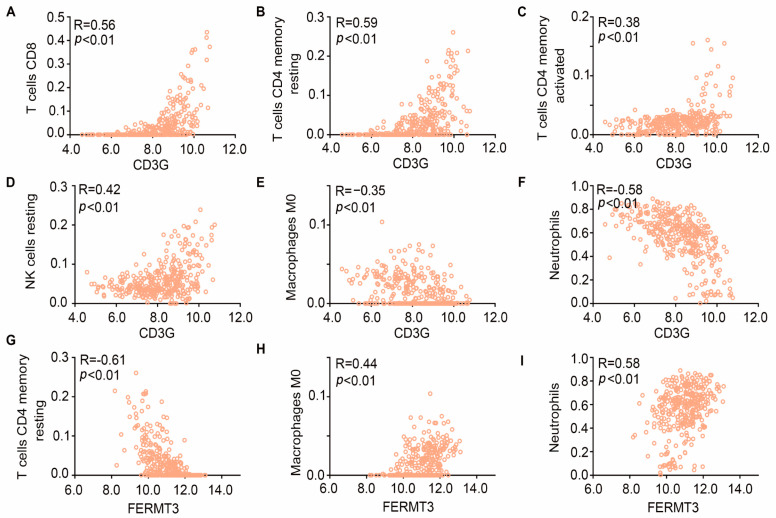
Correlation of hub genes versus immune cell infiltration. (**A**–**I**) The relationship between gene expression and immune cell infiltration levels was analyzed by Pearson co-expression analysis, including T cells CD8, T cells CD4 memory resting, T cells CD4 memory, NK cells resting, Macrophages M0, Neutrophils; R > 0.3 and *p* < 0.05 set as threshold.

Then, in order to investigate the link to the immune cells and two hub genes, we conducted correlation analyses based on the high and low expression levels of these hub genes. The observed relationship between gene expression and immune cell infiltration levels implied that the involvement of hub genes in the immune system response may play a role in regulating downstream cytokines. Specifically, the expression of *CD3G* exhibited a positive correlation with CD8 T cells, resting CD4 memory T cells, activated CD4 memory T cells, and resting NK cells, while it had negative relation with macrophages M0 and neutrophils. Furthermore, the expression of *FERMT3* demonstrated a positive correlation with M0 macrophages and neutrophils, while displaying a negative correlation with CD4 memory T cells (Figure 8A–I).

### 2.7. Sepsis Mice Model Construction and Cytokines Detecion In Vivo

**Figure 9:**
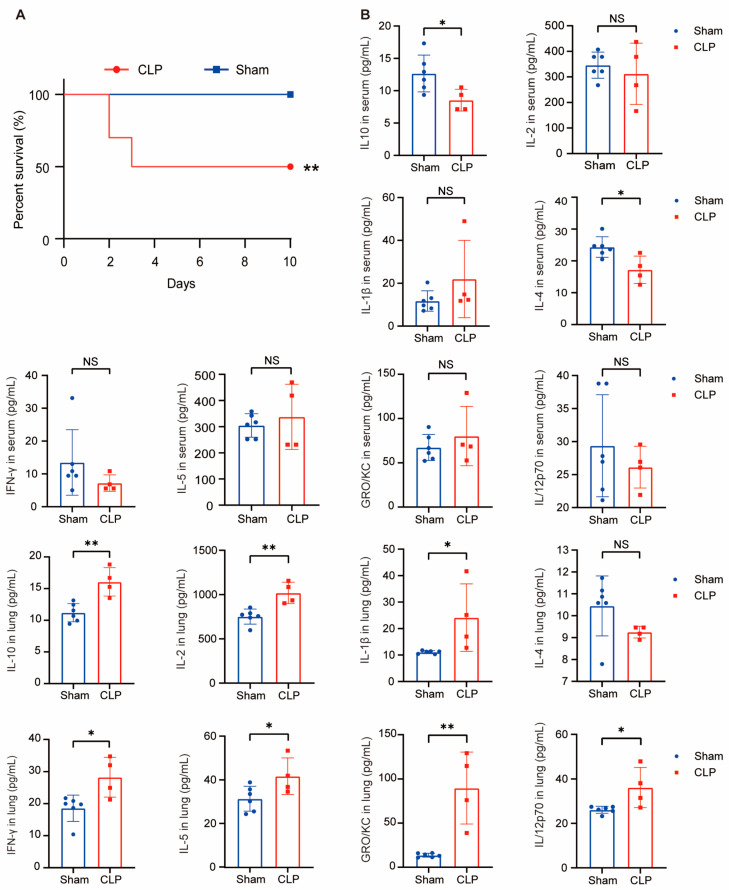
Inflammatory response in sepsis mice (log-rank test). (**A**) Survival rate in sepsis mice with sepsis and sham. (**B**) Levels of serum and lung homogenate cytokines. Luminex determination of serum IL-10, IL-2, IL-1β, IL-4, IFN-γ, IL-5, GRO/KC, IL-12p70 concentration in mice with sham (N = 6) and sepsis (N = 4). NS (not significant) *p* > 0.05, * *p* < 0.05, ** *p* < 0.01 vs. sham group.

To demonstrate the diagnosis function of hub genes in vivo, the sepsis mice model was established using the classic cecal ligation method. The sepsis mice began to exhibit mortality on the second day after surgery, resulting in a 10-day survival rate of 50%, while the sham-operated mice survived without any abnormalities, *p* < 0.01 (Figure 9A). The detection results of various cytokines in serum and lung tissue homogenate confirmed that the mouse model was consistent with sepsis [22,23]. Particularly, the levels of IL-10 and IL-4 in the CLP group’s serum samples were significantly lower than those in the sham group. (IL-10 in CLP group: 8.545 pg/mL vs. sham group: 12.66 pg/mL, *p* < 0.05; IL-4 in CLP group: 17.22 pg/mL vs. sham group: 24.35 pg/mL, *p* < 0.05). In the lung homogenates, the levels of IL-10, IL-2, IFN-γ, IL-5, GRO/KC, and IL/12p70 in the CLP group were significantly higher than those in the sham group. (IL-10 in CLP group: 16.07 pg/mL vs. sham group: 11.20 pg/mL, *p* < 0.001; IL-2 in CLP group: 1020 pg/mL vs. sham group: 752.2 pg/mL, *p* < 0.001; IL-1β in CLP group: 24.14 pg/mL vs. sham group: 11.11 pg/mL, *p* < 0.05; IFN-γ in CLP group: 28.48 pg/mL vs. sham group: 19.88 pg/mL, *p* < 0.05; IL-5 in CLP group: 41.67 pg/mL vs. sham group: 31.35 pg/mL; *p* < 0.05; GRO/KC in CLP group: 89.70 pg/mL vs. sham group: 13.62 pg/mL; *p* < 0.001; IL/12p70 in CLP group: 36.16 pg/mL vs. sham group: 26.14 pg/mL; *p* < 0.05) (Figure 9B).

### 2.8. The Expression of FERMT3 and CD3G in Sepsis Mice

**Figure 10:**
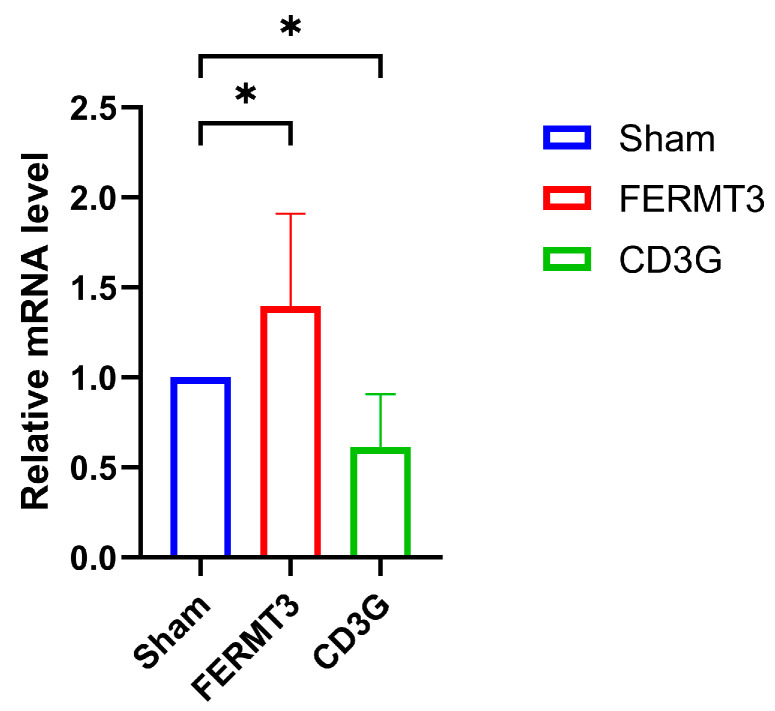
Validation of *FERMT3* and *CD3G* as a key biomarker of sepsis in the CLP group (N = 3) and sham group (N = 3). * *p* < 0.05 vs. sham group.

The expression of the two hub genes was evaluated using qRT-PCR in the serum of mice subjected to the CLP model. As shown in Figure 10, the expression of *FERMT3* in mice from the CLP model exhibited a significant increase (*p* < 0.05), while the expression of *CD3G* showed a significant decrease (*p* < 0.05) compared with the sham group, respectively. These findings align with the results obtained from the bioinformatic analysis.

### 2.9. CD3Gregulated the Regulation of Cytokines In Vivo

**Figure 11:**
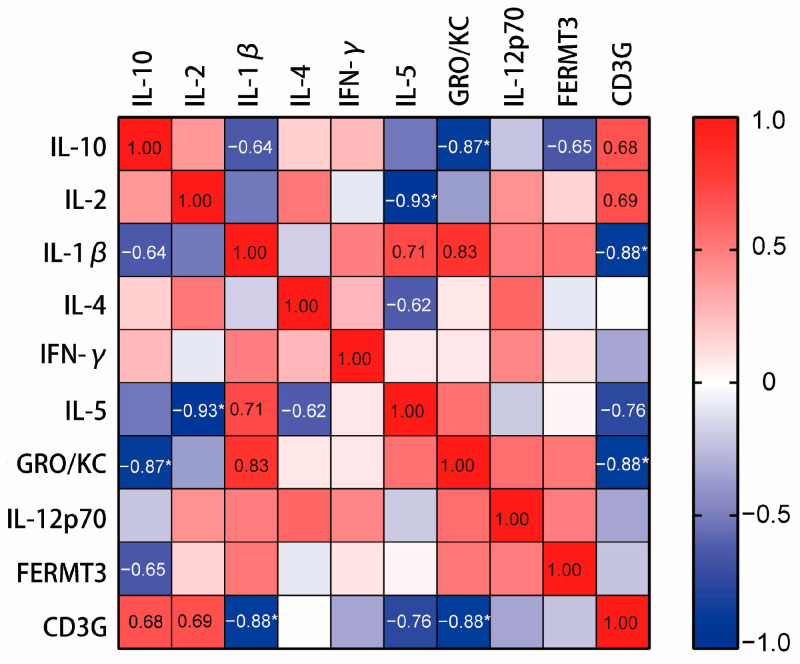
Correlation of serum cytokines levels with the hub genes of sepsis in the CLP group (N = 3) and sham group (N = 3). The correlation coefficient used for analysis is the Spearman correlation coefficient (r). Positive values of r indicate positive correlation, while negative values indicate negative correlation. The correlation was considered string if the value of r was between |0.6| to |0.79|, while the correlation was considered very strong if r > |0.79|. The range of the colors of the scale on the right, from blue to red, refers to r values from +1 to −1. * *p* < 0.05, vs. sham group.

Spearman correlation analysis was conducted to investigate the relationship between serum cytokines and the expression of hub genes in sepsis mice. The results indicated that the hub genes play a regulatory role in cytokines by influencing the inflammatory microenvironment. As shown in Figure 11, there is a significant negative correlation between the expression of *CD3G* and IL-1β as well as GRO/KC.

## 3. Discussion

Sepsis stands out as one of the most perilous infection diseases in clinical settings, contributing significantly to heightened morbidity and mortality among patients in the ICU. The effectiveness of sepsis diagnosis and prediction technologies plays a pivotal role, demanding meticulous attention. The primary challenge in sepsis therapeutics lies in how to enhance the specificity, sensitivity, and stability of diagnostic methods to effectively predict disease progression and guide clinical treatment plans [24]. In this study, we employed a novel approach by integrating bioinformatics analysis with clinical SOFA scores. SOFA scores represent the prevailing standard for diagnosing organ dysfunction, closely linked to the severity of sepsis [25]. Through this integrated approach, we successfully identified two immune-related upstream gene biomarkers, namely *FERMT3* and *CD3G*.

Specifically, *FERMT3* has been widely recognized for its role in cell adhesion, migration, and thrombosis. Several studies have demonstrated that mutations in *FERMT3* can lead to leukocyte adhesion deficiency syndrome type 3 (LAD3), resulting in defective platelet function and immunodeficiency [26,27,28]. These findings suggest a potential regulatory role of *FERMT3* in the interplay between inflammation and coagulation during the progression of sepsis. Furthermore, it has been confirmed that microthrombosis is closely associated with severe dysfunction and progression in sepsis. Sepsis patients with coagulation dysfunction, particularly those presenting with DIC, exhibit higher mortality rates [29,30]. In our study, bioinformatics analysis revealed a positive correlation between *FERMT3* and the SOFA score, and further confirmation showed a significant increase in *FERMT3* expression in CLP sepsis mice. *CD3G* is a key gene in the immune system, encoding a protein named CD3γ that is an important component of the T cell’s surface. It participates in the formation of T-cell surface TCR/CD3 complex and plays a crucial role in T-cell signal transduction. As a result, *CD3G* is closely related to the normal development, maturation, activation, and function of all T-cell subsets [31]. In our study, bioinformatics analysis identified a negative correlation between *CD3G* and the SOFA score, indicating severe dysfunction of T-cell immunodeficiency in severe sepsis patients with high SOFA scores. This relationship was further confirmed in a sepsis mouse model, where the expression of *CD3G* in CLP sepsis mice was significantly decreased compared with the sham group. Both *FERMT3* and *CD3G* showed a strong correlation with M0 macrophages and neutrophil cell infiltration, exhibiting positive and negative correlations, respectively. Based on these results, it can be reasonably inferred that *FERMT3* and *CD3G* may serve as potent biomarkers for specifically diagnosing and predicting the severity and progression of sepsis.

In addition, the cytokine storm is an important factor that exacerbates the course of sepsis, characterized by radical pro-inflammatory and anti-inflammatory responses, both of which contribute to immune disorders [32]. Several cytokines have been utilized as biomarkers to aid in sepsis diagnosis [23,33]. IL-1β, a crucial pro-inflammatory cytokine in response to infection, impacts nearly all types of cells [34,35]. An increase in the level of IL-1β in non-survivors suggests a correlation between IL-1β expression and sepsis outcome [36]. IFN-γ, primarily produced by activated T cells and NK cells, serves as a crucial activator of macrophages and inducer of class II major histocompatibility complex (MHC) molecule expression. Abnormally elevated IFN-γ levels are frequently associated with various autoimmune and inflammatory diseases [37,38,39]. GRO/KC, a chemokine ligand 1 (CXCL1), acts as a chemotactic agent for various immune cells, especially neutrophils or other non-hematopoietic cells, guiding them to the site of injury or infection. It plays important roles in regulating immune and inflammatory responses [40,41]. To further validate the accuracy and potential of the hub genes *FERMT3* and *CD3G* in sepsis diagnosis, we conducted correlation analysis between the expression of these genes in sepsis mice and the levels of typical inflammatory cytokine in vivo. The results demonstrated a high correlation between the expression of *CD3G* and typical pro-inflammatory cytokines in the sepsis mice. Specifically, the expression of *CD3G* is significantly negatively correlated with IL-1β and GRO/KC.

In the examination of cytokine levels in the serum and lung homogenate of sepsis mice, quantitative detection results revealed potential challenges associated with utilizing downstream molecules, particularly critical anti-inflammatory cytokines, such as IL-4, IL-10, etc. [11,42]. These downstream molecules, when used as biomarkers, may face interference from the complex internal regulatory environment of organisms, and this interference significantly impacts the sensitivity and consistency of results, potentially leading to the omission of crucial information. Furthermore, as downstream executor molecules, cytokine expression may vary in vivo based on the severity of the condition and the immune function status, whether extremely suppressed or hyperactive, of septic patients. In this investigation, a quantitative analysis of typical cytokines in both serum and lung homogenate was conducted. The results indicated that more sensitive data were obtained from lung homogenate, probably due to the substantial infiltration of immune cells into target tissues during sepsis. However, when using serum samples as commonly employed in clinical practice, the results showed significant individual differences in mice, suggesting apparent interference and variation which weakens their efficacy for clinical sepsis diagnosis and treatment. This observation aligns with clinical studies indicating reduced production of both proinflammatory and anti-inflammatory cytokines [22]. Ertel et al. also documented that the production of TNF α, IL-1 β, and IL 6 from patients with sepsis was less than 10–20% of that found in patients without sepsis, as evidenced by stimulating whole blood from patients with and without sepsis with endotoxin [43]. Therefore, considering gene expression as the upstream molecule of the signaling regulatory pathway proves to be a more stable and suitable biomarker for disease diagnosis and prognosis. Moreover, qRT-PCR demonstrates its value as a tool in establishing disease-specific biomarkers in clinical settings due to its accuracy, speed, and relatively straightforward process, taking about 6 h from sample preparation to obtaining results [44].

However, it is important to acknowledge the limitations and deficiencies of this study. The primary concern lies in the lack of further validation studies involving manipulation of these two hub genes both in vitro and in vivo, which would confirm the correlation between *CD3G* and *FERMT3* with downstream events related to cytokines and chemokines. This aspect should be addressed in further endeavors. Additionally, it is necessary to expand the relatively small dataset obtained from public databases. Furthermore, incorporating other pivotal indicators of sepsis alongside SOFA scores into the bioinformatics analysis could significantly enhance the accuracy and applicability of this model.

Briefly, we constructed a comprehensive model for screening sepsis-related genes, confirming that *FERNT3* and *CD3G* are DEGs in a mouse sepsis model. This suggests a close relationship between inflammation and coagulation, warranting further exploration to predict the severity of sepsis and identify suitable targets for treatment.

## 4. Materials and Methods

### 4.1. Experimental Design and Process

**Figure 12:**
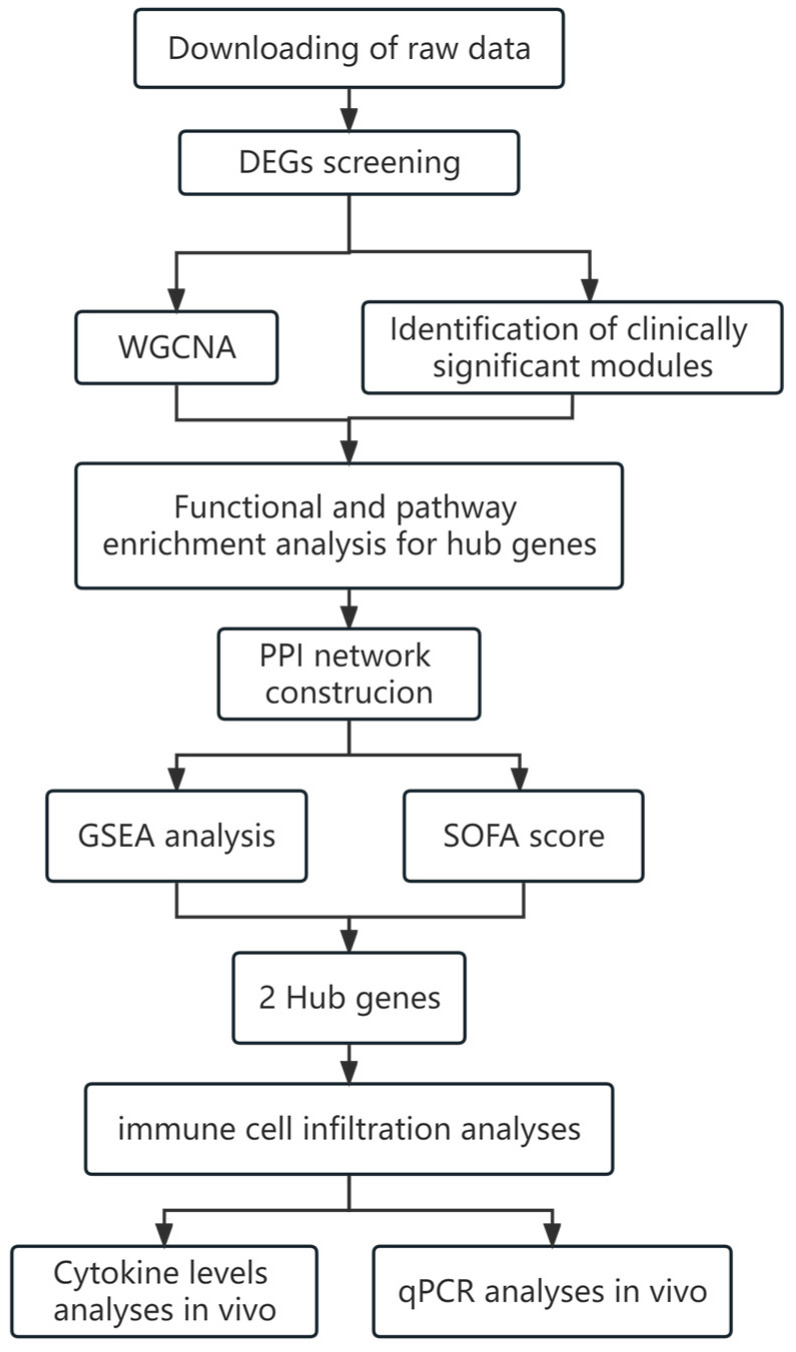
Overall design flow chart of the bioinformatic analysis and validation.

The flow chart of this study is shown in Figure 12.

### 4.2. Data Processing and Identification of DEGs

The RNA sequence and patient metadata, including sex, clinical severity measures, and outcomes, were obtained from GSE185263, and included peripheral blood specimens from patients with sepsis (n = 348) and healthy controls (n = 44). Relevant clinical traits were accessed from the NCBI Gene Expression Omnibus (GEO) database (https://www.ncbi.nlm.nih.gov/gds accessed on 10 February 2023). Baghela, et al. contributed the data which were acquired from Illumina NexSeq 200 (Homo sapiens; GPL16791) [45]. Before conducting WGCNA, the gene expression data underwent the following preprocessing steps: (I) probes were annotated and translated into gene names by merging information from the GPL16791 platform; (II) probes with null value were removed; (III) expression values of the same genes were merged and averaged; (IV) genes with a mean expression level < 0.5 were removed; (V) the MAD algorithm was employed to screen out genes with the top 4188 variation degrees, and these genes were subjected to WGCNA [46].

### 4.3. Differentially Expressed Genes (DEGs) Screening

EdgeR package in R software (version: 3.5.2) was used to analyze DEGs, comparing two groups of individuals. DEGs (|LogFC| > 1 and adjusted *p*-values < 0.05) were identified and plotted in volcano plots and in a heat map. Then, the real DEGs were obtained and included in the WGCNA analyses.

### 4.4. Weighted Gene Co-Expression Network Analysis

The R package ‘WGCNA’ was used to construct a co-expression network for the real DEGs. To construct the WGCNA, all genes from the 348 peripheral blood specimens, along with their corresponding clinical information, were imported into R (Version: 4.0.2). After filtering and trimming the raw expression data and clinical information, Pearson’s correlation analysis was performed to calculate the relationship between all the gene pairs. The results of this analysis were used to construct a weighted adjacency matrix. The threshold for identifying outlier samples was set at a cut height of 80. To attain a scale-free co-expression network, a matrix of similarity was developed using a soft power of β = 14. A scale-free network is characterized by a node degree distribution that adheres to a power law. It is distinguished by a small number of highly connected nodes, with the majority interacting with only a few neighbors, resulting in high robustness to random failure. In scale-free networks, the correlation between genes signifies their connectivity, with the average connectivity representing the mean of the total connectivity of all genes [47]. Furthermore, to estimate its connectivity property in the network, the weighted adjacency matrix was transformed into a topological overlap matrix (TOM) measure. Subsequently, the dynamic tree-cutting algorithm was used to define modules by cutting the clustering tree into branches. The identified modules were then assigned different colors for enhanced visualization.

### 4.5. Identification of Clinically Significant Modules

The relationships were examined between different module eigengenes and patient characteristics, including septic state, outcomes (improved for red, no improvement for white, and missing value for grey), gender, SOFA score, and age, using Pearson’s correlation analyses. Modules were deemed clinically significant if they exhibited a correlation with clinical characteristics (r > 0.3 and *p* < 0.05). The module eigengene (ME) was used to describe the expression pattern of each module in individual samples, while MM was measured by analyzing the correlation between the ME and the credibility within a module. GS was calculated based on the relationship between each gene and the characteristics of the clinical state of patients. Gene modules demonstrating a significance level of *p* < 0.05 between GS and MM were identified as clinically relevant modules, subsequently undergoing further analysis. Finally, genes meeting the criteria of MM > 0.8 and GS > 0.2 were designated as module core genes. These module core genes then interacted with DEGs, resulting in the identification of 333 genes for further consideration.

### 4.6. Functional Enrichment Analyses

To explore the biological functions closely associated with module core genes, GO and KEGG enrichment analyses were performed using the online tool KOPAS (http://kobas.cbi.pku.edu.cn/ accessed on 10 February 2023). The threshold for statistical significance was set at *p* < 0.05. The results of GO and KEGG analyses were visualized using the Bioinfo Intelligent Cloud tool (http://www.ehbio.com/Cloud_Platform/front/#/ accessed on 10 February 2023).

### 4.7. Construction of PPI Network and Identification of Hub Genes

PPI networks contribute to interaction information, which facilitates the identification of hub genes. We used the STRING database (http://www.string-db.org/ accessed on 10 February 2023) to construct a PPI network of the module core genes. Genes with a minimum required confidence score of ≥0.4 were set as significant within a full network model and then visualized using Cytoscape (http://www.cytoscape.org accessed on 10 February 2023). The Cytohub plugin was employed to select the top 20 genes with the highest degree scores in the network and then visualize them.

### 4.8. Gene Set Enrichment Analysis Construction

Pearson’s correlation analysis was performed to determine the relationship between the expression levels of hub genes and the SOFA scores in sepsis patients. Statistical significance was set at *p* < 0.05. Then, the hub genes underwent GSEA; GSEA (https://software.broadinstitute.org/gsea/index.jsp accessed on 10 February 2023) was performed to determine the biological processes that were enriched in the gene rank derived from DEGs between the two groups [13]. Enriched terms with false discovery rate (FDR) < 0.05 were significant.

### 4.9. Evaluating the Immune Cell Infiltration

The CIBERSORT algorithm (https://cibersort.stanford.edu/ accessed on 10 February 2023) and LM22 signature matrix were applied for estimating the relative abundances of 22 types of immune cells in each sample, based on the extracted gene expression data. CIBERSORT uses Monte Carlo sampling to obtain an inverse fold product *p*-value for each sample. Only samples with *p*-values < 0.05 were considered to be accurate immune cell fractions. The sum of the 22 immune cells’ proportions in each sample was 100%. The relationship between gene expression and immune cell infiltration levels was analyzed via Pearson co-expression analysis, while R > 0.3 and *p* < 0.05 were set as threshold.

### 4.10. In Vivo Validation Experiment of Animal Models

#### 4.10.1. Sepsis Mice Model

C57BL/6J mice (Charles River Laboratory Animal Technology Co., Ltd., Beijing, China) aged 8–12 weeks and weighing 20–25 g, were used for the animal model study. The mice were housed in specific pathogen-free rooms under 12 h light/12 h dark conditions, maintaining an ambient temperature of 26 °C ± 1 °C. All animal experiments were approved and supervised by the Institution of Animal Care and Use Committee at the Academy of Military Medical Science (IACUC-AMMS, Beijing, China).

Eighteen mice were randomly divided into two groups: the sham surgery group and the CLP (cecal ligation and puncture) group. The CLP operation was performed as previously described [48]. All mice were preoperatively fasted for 12 h. Subsequently, the mice were anesthetized and fixed on the operating table in a supine position. Under sterile conditions, a longitudinal incision was made in the abdominal skin and subcutaneous tissue to expose the cecum. The cecum of the CLP mice was ligated at a designated location (1 cm away from the distal end of the cecum), and the contents of cecum were pushed towards the distal end using a cotton swab. Then, two punctures of the cecum were performed using a 20 sterile needle at the distal end of the ligation, followed by routine abdominal closure. The sham surgery group did not perform cecal ligation and puncture operations, but directly closed the abdomen. All groups were monitored for 10 days to calculate their survival rate [49]. The pathological structure of the lung, liver, and kidney tissues in sham and CLP mice was analyzed using histopathologic examination (HE). The HE stainings of lung, liver, and kidney in sham and CLP groups are shown in Figure S1. Survival curve, pathological structure, and measurements of inflammatory cytokine were utilized to evaluate the sepsis mice models.

#### 4.10.2. qRT-PCR Detecion of FERMT3 and CD3G Gene Expression Levels

At 10 days after surgery, total RNA from the serum of mice in both the sham and CLP groups was extracted using FreeZol Reagent (Nanjing Vazyme Biotech Co., Ltd., Nanjing, China). Reverse transcription was carried out using HiScript III All-in-one RT SuperMix Perfect for qPCR (Nanjing Vazyme Biotech Co., Ltd.). Taq Pro Universal SYBR qPCR Master Mix (Nanjing Vazyme Biotech Co., Ltd.) was used to amplify gene expression in order to perform the real-time quantitation. The primers employed in the study were as follows:

5′-CAGGCTGACCTCTGAAGGAA-3′ (*FERMT3* forward primer),
5′-GGCTGTTTCTGCTAGCCTG-3′ (*FERMT3* reverse primer),
5′-CTTCTGACTTGTGGCTTGACTGAC-3′ (*CD3G* forward primer),
5′-CCTTTGCTCCTTGACACTGATACG-3′ (*CD3G* reverse primer),
5′-ACGGCAAATTCAACGGCACAG-3′ (*GAPDH* forward primer),
5′-ACACCAGTAGACTCCACGACATAC-3′ (*GAPDH* reverse primer).

*GAPDH* was used as reference to determine loading controls. The 2^−ΔCT^ method was used to calculate the relative expression level.

#### 4.10.3. Inflammatory Cytokine Measurements

The inflammatory cytokines in both serum and lung were assessed 10 days after surgery. Blood samples were obtained into Eppendorf tubes between 9:00 and 11:00 a.m. following an overnight fast. After 1 h storage at room temperature, the tubes were centrifuged at 3000 rpm/min at +4 °C for 20 min within 12 h. The resulting serum was then stored at −80 °C. Lung tissues were homogenized in physiological saline using a tissue homogenizer. Cytokines and chemokines, including IL-10, IL-2, IL-1β, IL-4, IFN-γ, IL-5, GRO/KC, IL-12p70 were detected using the BIO-RAD panel (Hercules, CA, USA) and the Luminex X-2000, following the manufacturer’s guidelines. Milliplex Analyst 5.1 software (EMD Millipore, Billerica, MA, USA) was employed with the 4-parameter logistic (4-PL) model. This model optimizes accuracy and precision across the maximum usable calibration range, facilitating the calculation of cytokine concentrations in both serum and lung homogenate. All samples were processed in duplicate.

#### 4.10.4. Correlation Analysis on the Hub Genes and Cytokines

Spearman’s correlation analysis was performed to determine the relationship between the expression levels of hub genes and the cytokines in serum of sepsis mice.

#### 4.10.5. Statistical Analysis

The normality distribution of the data was tested with the Shapiro–Wilk test and the data were expressed as mean ± standard deviation (SD). Comparisons between two groups were performed using *t*-tests. Survival results were analyzed using the log-rank test. One-way analysis of variance with Dunnett’s multiple comparisons test was used to determine the differences of genes in multi-groups. Values of *p* less than 0.05 were considered statistically significant.

## 5. Conclusions

In summary, the present study successfully constructed a bioinformatics analysis method incorporating SOFA scores to characterize the severity of sepsis. Two hub genes, *FERMT3* and *CD3G*, were identified, each playing important roles in inflammation, thrombosis, and T-cell function, respectively. The specific expression changes of these hub genes in sepsis mice were validated through qRT-PCR, consisting with the bioinformatics results. Furthermore, the correlation between these hub genes and downstream cytokines in sepsis mice was substantiated, suggesting that *FERMT3* and *CD3G*, as upstream molecules, may possess more stable characteristics for diagnosis compared with downstream cytokines. These findings implied that FERMT3 and CD3G probably play pivotal roles in the pathogenesis and progression of sepsis, offering potential as stable biomarkers for assessing organ dysfunction and predicting ICU mortality risk of sepsis, warranting further research.

## Data Availability

The data presented in this study are available on request from the corresponding author.

## Supplementary Materials

The following supporting information can be downloaded at: https://www.mdpi.com/article/10.3390/ijms25020749/s1.
