# Supplementary material for: Immune-Related Molecules CD3G and FERMT3: Novel Biomarkers Associated with Sepsis
Source: Int J Mol Sci. 2024 Jan 6;25(2):749. doi: 10.3390/ijms25020749 (PMC10815248; doi:10.3390/ijms25020749)

Supplementary Material

Figure S1: The HE staining of tissues in the sham and CLP groups at 10 days. (A) The lung tissues of the CLP group exhibited a significant infiltration of lymphocytes and neutrophils, (B) Hepatocellular degeneration surrounding liver blood vessels is observed in multiple areas, (C) Minor venous congestion and dilation were observed in kidney.

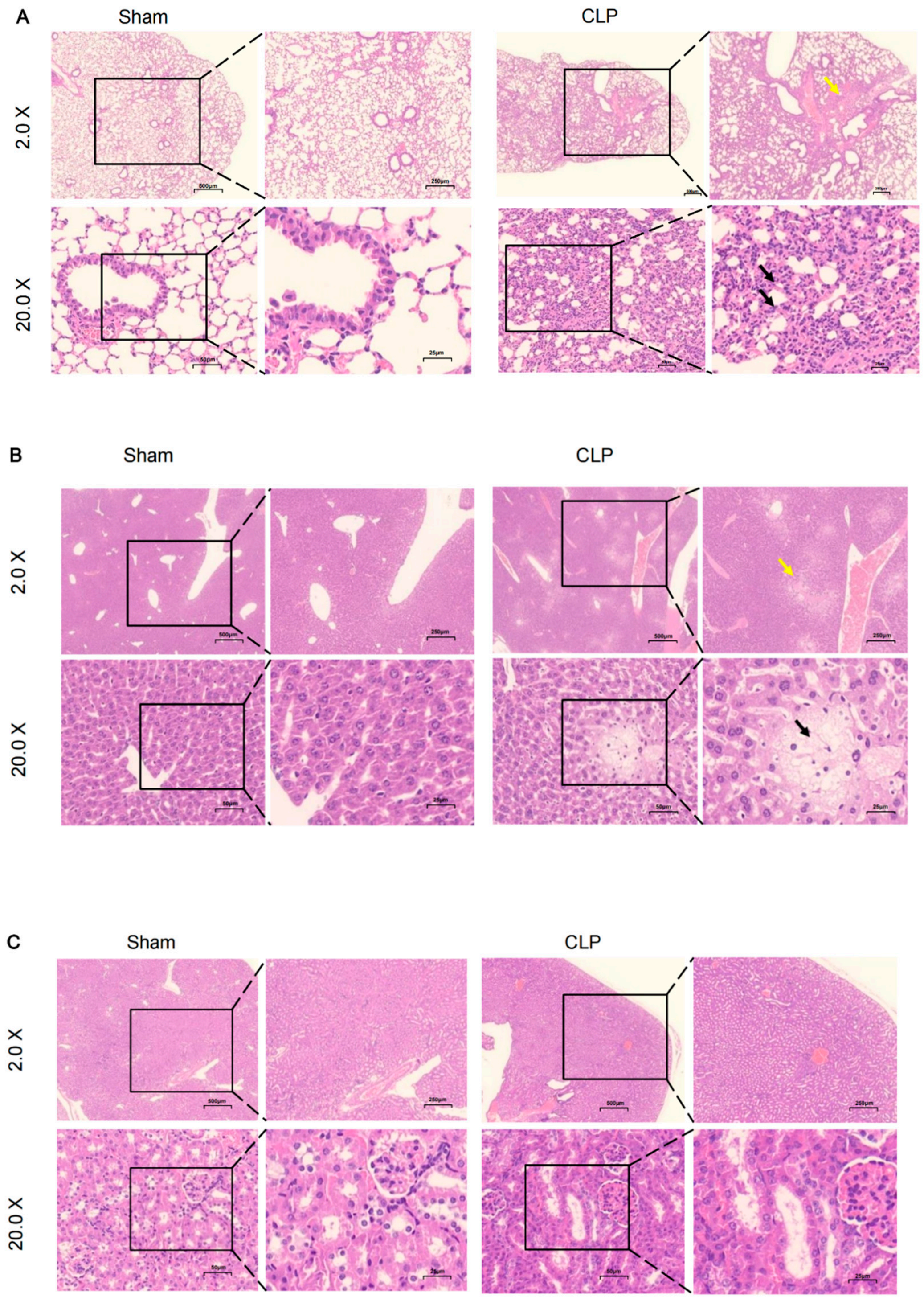

Figure S2: The heat map of top ten up-regulated and down-regulated genes, respectively.

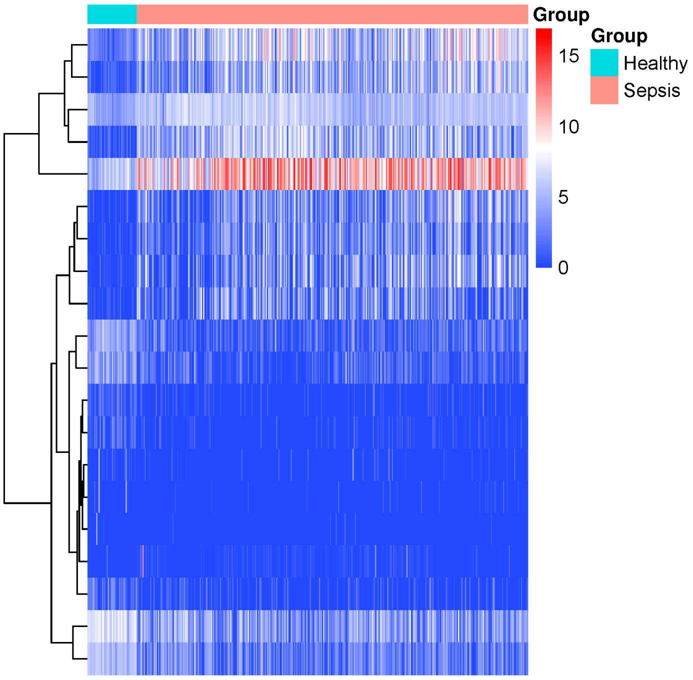

Supplement: Supplementary file 1 [file ijms-25-00749-s001.zip › ijms-2734527-supplementary.pdf]
